# High-throughput retrotransposon-based fluorescent markers: improved information content and allele discrimination

**DOI:** 10.1186/1746-4811-5-10

**Published:** 2009-07-28

**Authors:** Maggie Knox, Carol Moreau, James Lipscombe, David Baker, Noel Ellis

**Affiliations:** 1 Dept. Crop Genetics, John Innes Centre, Colney Lane, Norwich, NR4 7UH, UK; 2 The John Innes Centre Genome Laboratory (JGL), John Innes Centre, Colney Lane, Norwich, NR4 7UH, UK

## Abstract

**Background:**

Dense genetic maps, together with the efficiency and accuracy of their construction, are integral to genetic studies and marker assisted selection for plant breeding. High-throughput multiplex markers that are robust and reproducible can contribute to both efficiency and accuracy. Multiplex markers are often dominant and so have low information content, this coupled with the pressure to find alternatives to radio-labelling, has led us to adapt the SSAP (sequence specific amplified polymorphism) marker method from a ^33^P labelling procedure to fluorescently tagged markers analysed from an automated ABI 3730 *xl *platform. This method is illustrated for multiplexed SSAP markers based on retrotransposon insertions of pea and is applicable for the rapid and efficient generation of markers from genomes where repetitive element sequence information is available for primer design. We cross-reference SSAP markers previously generated using the ^33^P manual PAGE system to fluorescent peaks, and use these high-throughput fluorescent SSAP markers for further genetic studies in *Pisum*.

**Results:**

The optimal conditions for the fluorescent-labelling method used a triplex set of primers in the PCR. These included a fluorescently labelled specific primer together with its unlabelled counterpart, plus an adapter-based primer with two bases of selection on the 3' end. The introduction of the unlabelled specific primer helped to optimise the fluorescent signal across the range of fragment sizes expected, and eliminated the need for extensive dilutions of PCR amplicons. The software (GeneMarker Version 1.6) used for the high-throughput data analysis provided an assessment of amplicon size in nucleotides, peak areas and fluorescence intensity in a table format, so providing additional information content for each marker. The method has been tested in a small-scale study with 12 pea accessions resulting in 467 polymorphic fluorescent SSAP markers of which 260 were identified as having been mapped previously using the radio-labelling technique. Heterozygous individuals from pea cultivar crosses were identifiable after peak area data analysis using the fluorescent SSAP method.

**Conclusion:**

As well as developing a rapid, and high-throughput marker method for genetic studies, the fluorescent SSAP system improved the accuracy of amplicon scoring, increased the available marker number, improved allele discrimination, and was sensitive enough to identify heterozygous loci in F_1 _and F_2 _progeny, indicating the potential to develop high-throughput codominant SSAPs.

## Background

The SSAP marker method described by Ellis *et al*. [1] for pea assays insertion sites for *PDR1*, a Ty1-*copia *like retrotransposon found at about 200 copies per haploid genome. These SSAP markers have allowed the integration of *Pisum *genetic maps from different populations especially where there are common parents [1]. Many other transposable elements have been captured as markers for mapping and diversity analysis in a wide range of plant species including pea [1-4], barley [5], *Hibiscus *[6], potato [7], sweetpotato [8], cotton [9], agave [10], wheat [11], vine [12], *Vicia *[13], lettuce [14], cashew [15] and cucumber [16]. Though these studies all use SSAP markers, there are fundamental differences in the generation of the DNA template and subsequent amplification. In many of these cases [4-16] the SSAP approach was AFLP-like [17] in that it reduced amplicon complexity with a double restriction enzyme digest, using a frequent and a rare cutting enzyme, followed by the appropriate adapter ligation. PCR amplification was then carried out in two stages: first a pre-amplification with the adapter based primers and limited base selection, followed by a re-amplification with a labelled specific primer and one adapter primer with additional bases of selection. The majority of marker amplicons produced were generally in the 50 – 500 base-pairs (nt) range.

For pea the SSAP marker method, based on *PDR1 *the relatively low copy number Ty1-*copia *– like retrotransposon insertions [1,2] or on the high copy number transposable elements *Pi*s*1 *and *Cyclops *[3,4,18], has been used for mapping and diversity analysis. This method involves a single restriction digest and adapter ligation, and requires no pre-amplification. This approach therefore does not involve an enzyme digestion based complexity reduction as in AFLP [17]; it is a multiplexed, manual, ^33^P labelled, PAGE (polyacrylamide gel electrophoresis) system with marker amplicons in the range ca. 100 – 1300 nucleotides (nt) that appears to be suitable for conversion to automation and fluorescent amplicon detection.

To enable cross-referencing of existing SSAP markers between the radio-labelled method and fluorescent approaches, the range of amplicon sizes resulting from both techniques needed to be the same, and the correspondence between the band pattern from ^33^P PAGE and fluorescent peaks needed to be established. Here we describe the conversion from a manual radio-labelling method to a high-throughput automated system, the capture of *PDR1 *retroelement related fluorescent SSAP markers in the range 100 – 1300 nt and their use in pea genetics. We also describe the development of codominant markers using the analysis of fluorescent peak areas to calculate dosage ratios for both F_1 _and F_2 _individuals, and discuss the potential use of these markers with their improved information content.

## Results and discussion

### Fluorescent SSAP marker development

**Figure 1:**
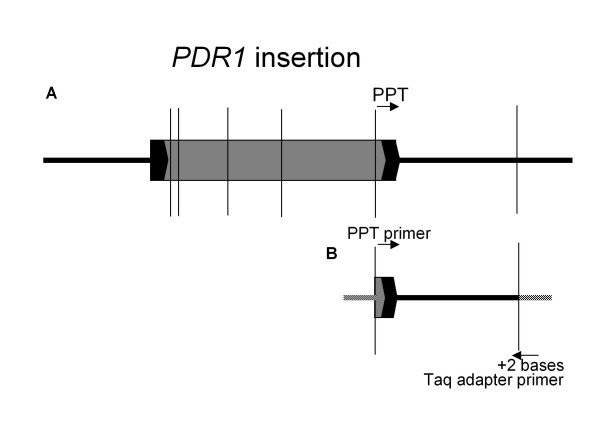
**Schematic of a *PDR1 *insertion in the pea genome and template for PCR**. **A**. The *PDR1 *insertion (grey area with black blocked termini) within genomic DNA (thick black horizontal lines), with the *Taq*I sites (thin vertical lines) shown. The *PDRI *element first sequenced (X66399.em.pl) has five known *Taq*I sites (vertical lines). A sixth *Taq*1 site external to the element is shown. **B**. The distance to an external site differs from one *PDR1 *insertion site to another; the Taq adapter is shown (chequered line) at this location. PCR is carried out with the labelled PPT (polypurine tract) – specific primer of the element, which is adjacent to the LTR (long terminal repeat – black blocked termini with chevron), and the Taq adapter primer with two bases of selection on the 3'-end. A range of amplicon sizes are obtained dependent on the position of *Taq*I sites 3' to the element insertion within the pea genome.

The most common SSAP [1] method in pea uses a *Taq*I restriction digest, with corresponding adapter ligation, followed by PCR amplification using a specific primer, and a Taq adapter primer. To capture *PDR1 *insertions conveniently it is necessary to have two bases of selection at the 3'-end of the Taq adapter primer (Figure 1). In all there are 16 possible primer combinations generating amplicons in the range ca. 100 – 1300 nt. The ^33^P radio-labelled SSAP method exploits the phosphorylation of the 5'-end of the sequence-specific primer in a kinase reaction. For the automated system a fluorescent tag, 6-FAM or HEX, was attached at the 5'-end of the sequence-specific primer during its synthesis (SIGMA-ALDRICH). Fluorescently labelled primers were HPLC-purified to generate a homogeneous length distribution [19].

Initial attempts to generate fluorescent SSAP markers merely substituted an HPLC-purified 6-FAM labelled PPT specific primer (Table 1) for the ^33^P PPT specific primer described previously [1]. When tested on the ABI3730 *xl *this experiment gave consistent but unacceptable results regardless of the amplicon dilution: the labelled primer peak was vast and its intensity overwhelmed that of the amplicon peaks, which were low. There was no obvious correspondence between the peak pattern and those previously observed from the ^33^P PAGE method and the expected amplicon size range was not achieved.

**Table 1:** Adapter and primer details

Primer	Sequence
ds Taq adapter	5'- ATGAGTCCTGAA-3' 3' – TACTCAGGACTTGC-5'

PPT	5'- [^6FAM ^or ^HEX ^or neither] ATTCACCAGCTTGAGGGGAG-3'

Taq adapter +2 [16 combinations]	ATGAGTCCTGAACGANN^a^

^a ^NN two bases of selection

To overcome these problems a ^6FAM^PPT primer without HPLC-purification was tested and modifications to primer concentrations [1] were made. A range of primer concentrations was tested in a duplex PCR which contained the specific ^6FAM^PPT primer and the Taq+2 primer, and also a triplex of primers that consisted of the duplex mix plus unlabelled PPT primer. The peak patterns from a dilution series (1/10 – 1/80) of the amplicons from both of the duplex and triplex PCRs were compared.

Results from the duplex reaction were variable with respect to peak intensity and SSAP amplicons > 900 nt were not resolved, or were of very low intensity, barely discernible above the background. However, triplex primer reaction conditions containing ^6FAM^PPT (not HPLC purified), unlabelled PPT, and the Taq+2 adapter primer (each at 0.1 μM), at tenfold dilution of amplicons in formamide loading solution, resulted in a reproducible peak pattern that matched the manual ^33^P PAGE banding pattern. Peaks corresponding to bands in the expected range 100 to >1200 nt, were obtained. The fluorescent tag HEX was also tested and gave comparable results to 6FAM. Subsequent experiments used both tags with different primer combinations, where the PCR products were co-electrophoresed on the ABI3700 *xl*.

### Comparing ^33^P bands to fluorescent peaks

**Figure 2:**
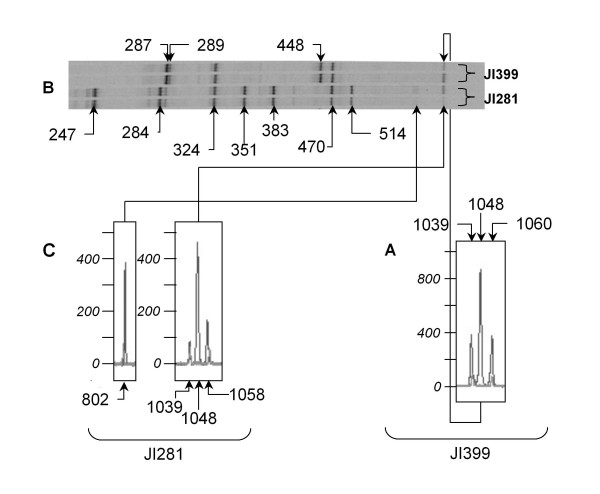
**^33^P band and fluorescent peak pattern comparison**. Fluorescent peak patterns with the triplex primer combination ^6FAM^PPT/PPT/Taq+TG, showing individual electropherograms for specific amplicons, **A **1039 – 1060 nt for JI399, **C **802 and 1039 – 1058 nt for JI281; the horizontal axis represents nt, and the vertical axis represents relative fluorescent units (RFU) [20]. The corresponding radio-labelled reactions with ^33^P PPT/Taq+TG for both accessions, shown replicated in **B**, covers a partial gel image from 247 to ca. 1100 nt. The band sizes (nt) were determined by virtue of their pattern of occurrence from the ^33^P gels and their corresponding fluorescent peak assessed with GeneMarker Version 1.6 software.

Peak and band patterns of 12 pea accessions from 16 primer combinations were compared and previously mapped markers identified. Band intensity differences between amplicons in the ^33^P PAGE procedure were mimicked by peak area variation with the fluorescent SSAP triplex primer method. Faint bands in the ^33^P procedure corresponded to low intensity fluorescence. For example the JI281 faint band at 802 nt on ^33^P PAGE (Figure 2B) corresponds to a relatively weak signal fluorescent peak reaching about 400 RFU (relative fluorescent units [20]) (Figure 2C). Strong bands corresponded to high intensity peak signals, but on occasion these were resolved into doublet or triplet peaks. These improvements to allele discrimination with fluorescence are obvious from the triplet of peaks at >1000 nt (Figure 2A and 2C) that appear as a single band on ^33^P PAGE in both JI281 and JI399 (Figure 2B). Peaks at 1039 and 1048 nt (Figure 2A and 2C) are monomorphic in both pea lines. The peak at 1060 nt for JI399 and 1058 nt for JI281 (Figure 2A and 2C) are polymorphic markers, putatively allelic, with mapping potential from the fluorescent method, but not scorable in the ^33^P SSAP method.

Fluorescent SSAP has already been described based on the double digest AFLP-like method [6,8] and captures marker amplicons of less than 600 nt. The method described here samples the whole genome more comprehensively as it is based on a single enzyme digest and so there is no complexity reduction step of template DNA. Furthermore this method requires no pre-amplification and the only dilution required was into the electrophoresis loading medium. The presence of both labelled and unlabelled specific primers (neither HPLC purified) presumably allows a competition reaction in the triplex primer PCR that helps to regulate peak intensities removing the need for additional dilution series. The need for pre-amplifications and dilution of PCR products adds steps for pipetting error and the use of consumable and manual resources. However, the method described here with the introduction of the unlabelled specific primer along with its fluorescent counterpart removed the need for these steps, kept the peak signal intensity within range for analysis and base calling by the software, and recovered amplicons in the expected size range.

### SSAP data and scoring accuracy

Marker scoring for 12 pea accessions with the fluorescent method yielded a total of 510 markers, 467 of which were scored as polymorphic. In comparison, using the same combination of selective bases, the manual ^33^P method yielded 352 markers, 318 of which were scored as polymorphic. The fluorescent method has made marker calling more accurate as it has the ability to detect a 1 nt difference between amplicons; this is much more difficult from manual PAGE so many potential polymorphic markers were left unscored.

The increased number of markers scored in the fluorescent system were mainly derived from closely bunched ^33^P marker bands which were resolved to multiple fluorescent peaks, as illustrated in Figure 2 for markers >1000 nt. Problems with band intensity variation and PAGE quality from the ^33^P method accounted for some of the marker number differences between the two methods. The correspondence between 260 mapped markers that appeared to be common to both methods was tested by examining their allelic distribution in a set of 12 diverse pea accessions. Of these the scores for 40 markers were found to differ between the two methods. Examination of the band and peak traces for these 40 markers showed that the differences in scores resulted from poor resolution from ^33^P PAGE. In 30 of the 40 cases a single ^33^P band resolved into two or more fluorescent peaks in close proximity. This was found for amplicons in the size range 200 – 1300 nt. The remaining ten differences between the two methods involved problems with band intensity and gel quality variation from the radiolabelled method which affected the accuracy of scoring. ^33^P bands corresponding to amplicons of similar size that are separated by some distance on manual PAGE may be difficult to score because of edge effects generating a 'smile'; this is not a problem with capillary electrophoresis. This problem of band identity becomes even more acute when the band signal is faint.

In this study we have found that for amplicons smaller than 1200 nt the estimated size range deviation was 0 to 0.5 nt between duplicate samples, monomorphic bands, and repeat ABI runs for the ^6FAM^PPT and ^HEX^PPT testing (run separately or together in the same capillary). The 0 to 0.5 range of accuracy was used throughout this study as an indication for the same marker peak. Where the peak corresponded to an amplicon >1200 nt (the upper extreme for the LIZ size standard) or had a weak fluorescent signal, size was determined using the manual calling facility of the software, but the deviation between duplicate samples was sometimes 1–2 nt; in these cases duplicate samples were found to be crucial for amplicon identification.

As a general observation an additional source of marker number differences between manual PAGE and the fluorescent system involves a human bias and selection procedure of allele scoring from ^33^P PAGE: polymorphic markers that appear difficult to score accurately would tend not to be attempted. The base calling and binning of fluorescent markers removes this element of human bias.

Errors in manual scoring and data tabulation create problems for genetic mapping and further data analysis [21,22]. The binning of peak data to a spreadsheet that is easily copied to a MS^® ^Excel file from the GeneMarker Version 1.6 software (SoftGenetics LLC^®^) reduces data error. Manual peak calling and the binning facilities of the software were found to be useful where GeneMarker failed to call a peak; this was found necessary where the signal for a peak was below the set detection threshold, or where there was a run of close peaks that differ by one to two nt, and for peaks greater than 1200 nt (the upper limit of the LIZ size standard). Even with the benefits of the software all peaks needed to be checked manually.

The major aim of this study was to develop and optimise the well established SSAP marker method from ^33^P manual PAGE as a high-throughput fluorescence system.

Since its inception [1] the *PDR1 *SSAP marker method using ^33^P manual PAGE has provided a core set of markers for pea genetic analysis and comparative mapping, but the fluorescent marker method provides an increased marker number and more information is associated with each marker. The use of fluorescence has eliminated many of the problems discussed above, and has made the scoring of markers all the more accurate.

### Identity of fluorescent amplicons

A major requirement in the development of the fluorescent SSAP method was the need to establish the correspondence between the ^33^P banding pattern and fluorescent peaks. This was an important criterion as these ^33^P markers constitute a reference set of markers for pea map integration.

With the fluorescent SSAP method amplicons are identified on the basis of size in relation to an incorporated known size ladder which is present in every sample. Amplicon size variation may be the consequence of conformational polymorphism from single base differences. The SSCP (single strand conformational polymorphism) method is a recognised marker method [23,24] and an example of SSCP was encountered during the course of *PDR1 *SSAP marker development [25,26]. A codominant marker pair, distinguishing JI15 and JI399 (two mapping parental lines) migrated on manual PAGE as if they differed in size. Both were sequenced and found to be 277 nt in length [26]. These markers exhibited conformational polymorphism as a consequence of a single base substitution and two independent indels. These two codominant alleles behaved in the same manner when run in the fluorescent system.

One minor disadvantage of the fluorescent system is the difficulty isolating fluorescent amplicons for further marker characterisation and development. Almost certainly there will be the requirement to have sequence information of specific amplicons and the need either to revert to ^33^P PAGE for band extraction, or whole scale cloning of the amplification products. These methods have been explored [25] for the development of the *PDR1 *retroelement insertion sites as a codominant marker system described as RBIP (retrotransposon based insertional polymorphism) [27]. During the development of the RBIP markers many of the *PDR1 *right and left-hand flanking regions from the ^33^P SSAP have been extracted from PAGE gels and sequenced [26].

### Peak area and heterozygote identification

**Figure 3:**
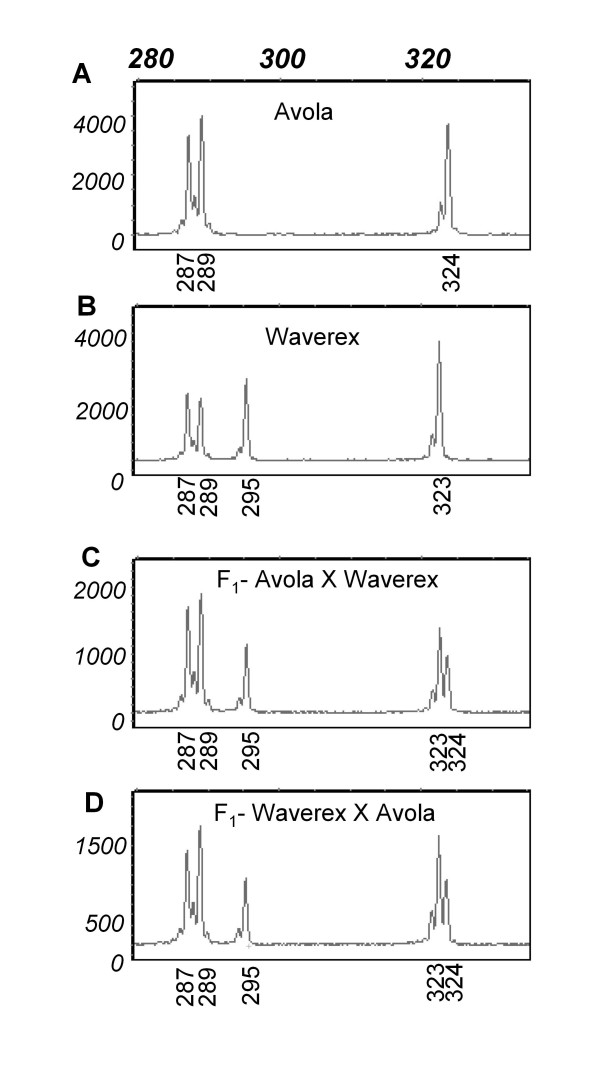
**Behaviour pattern of five loci from two pea cultivars and their reciprocal F_1 _progeny**. Electropherogram output in the range 280 – 330 nt showing an expanded scale to emphasis the peak differences, from the triplex SSAP using primer combination ^HEX^PPT/PPT/Taq+TG: **A **Avola, **B **Waverex, **C **F_1 _hybrid Avola × Waverex, **D **F_1 _hybrid Waverex × Avola. The pollen donor parent for **C **and **D **is the second name of the cross. Vertical and horizontal axes are described in Figure 2.

SSAP markers from ^33^P PAGE are dominant and so have low information content for analysing F_2 _populations [3]. Fluorescent SSAP markers have the potential to be codominant. To test this we examined the behaviour of the fluorescence approach with some F_1 _hybrids. Figure 3(A – D) shows the behaviour of alleles at five loci where the amplicon products were in the range 280 – 330 nt for the cultivars Avola and Waverex as well as their reciprocal F_1 _hybrids. Of these loci, two were monomorphic (Figure 3A–D, peaks at 287 and 289 nt) and so are homozygous in the F_1 _hybrids, while the other three were polymorphic and heterozygous (in fact hemizygous) for the insertion site. The Waverex peak at 295 nt (Figure 3B), absent in Avola (Figure 3A), was present in both F_1_s from the reciprocal crosses (Figure 3C and 3D). For the peaks at 323 nt from Waverex and 324 nt from Avola (Figure 3A and 3B), both F_1 _individuals carried both alleles (Figure 3C and 3D). If peak area indicates the dosage of the insertion site then these heterozygous alleles should be identifiable as having a reduced peak area with respect to the homozygote.

**Figure 4:**
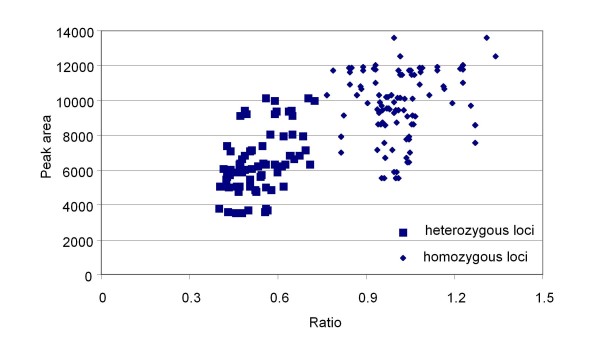
**Scatterplot of peak area against ratio for F_1 _individuals**. 168 data points (72 polymorphic and 96 monomorphic values) from 12 F_1 _individuals normalised against two pairs of monomorphic peaks to obtain the ratios.

Ratiometric data analysis [see Additional file 1] showed that the heterozygous amplicons had a reduced peak area, approximately a half that of the corresponding homozygous amplicons (Table 2). Ratiometric analysis of these peak areas showed clearly that heterozygous alleles could be detected (Figure 4) and distinguished from the homozygotes. These F_1 _data show that there is a ratio range within which the two classes fell, 0.4 to 0.72 for the heterozygote and 0.76 to 1.34 for the homozygote class (Figure 4). This range variation will need to be determined empirically for individual markers in different experiments.

**Table 2:** Mean peak area ratios in F_1 _zygosity tests

	Homozygote				Heterozygote					
					Normalised to m1/m2			Normalised to m3/m4		
Peak	m1	m2	m3	m4	p1	p2	p3	p1	p2	p3
Size (nt)	287	289	448	470	295	323	324	295	323	324
aw/a^†^	1.0 (0.03)	1.0 (0.03)	0.84 (0.02)	1.23 (0.03)			0.55 (0.07)			0.44 (0.03)
aw/w^†^	0.98 (0.04)	1.01 (0.03)	1.08 (0.03)	0.91 (0.03)	0.51 (0.03)	0.53 (0.08)		0.6 (0.03)	0.62 (0.07)	
wa/a^†^	0.99 (0.03)	1.02 (0.04)	0.83 (0.04)	1.25 (0.06)			0.53 (0.03)			0.43 (0.02)
wa/w^†^	0.97 (0.04)	1.03 (0.04)	1.06 (0.06)	0.95 (0.05)	0.46 (0.03)	0.55 (0.06)		0.55 (0.07)	0.66 (0.04)	
**Mean (SD) of 48**		**1.04** **(0.04)**		**1.02** **(0.16)**		**Mean (SD) of 36**	**0.52** **(0.06)**		**Mean** **(SD) of 36**	**0.55** **(0.1)**
			**Mean (SD) of 96**	**1.01** **(0.11)**					**Mean** **(SD) of 72**	**0.54** **(0.08)**

^† ^a = Avola; w = Waverex; aw = Avola × Waverex; wa = Waverex × Avola

A blank cell indicates no value as the peak was absent. The raw peak area values from the F_1 _data in Additional file 1 were first normalised with respect to the sum of the peak areas of the monomorphic pairs m1/m2 and m3/m4 and then the ratio of these normalised values was determined with an expected mean of 1 for the homozygote and 0.5 for the heterozygote. Values for each class show the mean from six F_1_s with the SD in brackets. Collective means and SD for both the homozygote and heterozygote classes can be seen in the bottom two rows [see Additional file 1 for all data input to this Table].

### Heterozygote differentiation within F_2 _individuals

The extent to which the ratio values for homozygous and heterozygous classes vary within an F_2 _population was examined.

**Figure 5:**
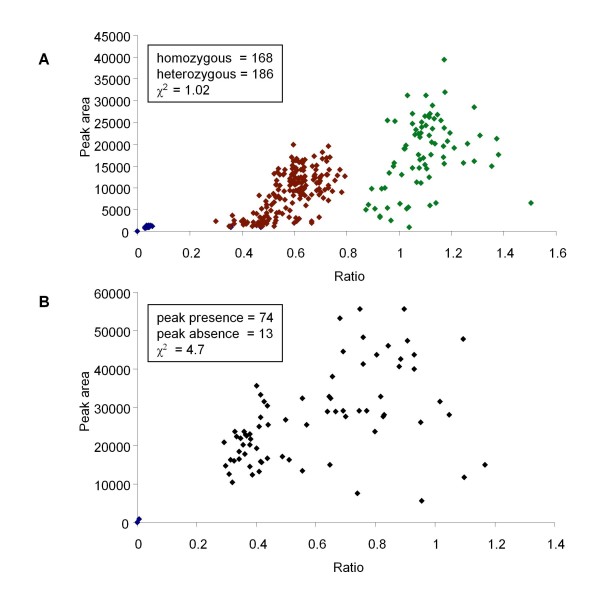
**Scatterplot of peak area against ratio for F_2 _individuals**. Data normalised against one pair of monomorphic peaks to obtain the ratios from an F_2 _population: **A**. 354 data points with four polymorphic fluorescent SSAP markers, showing the data points from the three classes: homozygous peak absent (blue), the heterozygous (red), and the homozygous peak present (green); **B**. 87 data points with one polymorphic marker.

Four polymorphic fluorescent SSAP markers (261, 274, 307, and 396 nt; Table 3), and their respective peak areas from F_2 _individuals were used to calculate peak area ratios, normalised against two monomorphic peaks as described for the F_1_s [see Additional file 1]. Figure 5A shows the plot of ratios against raw peak values for the four markers, and represents the collective 354 data points out of a possible 376 (12 failed PCRs). The plot shows a clear distinction between the 168 homozygous and 186 heterozygous classes with a good fit to the expected 1:1 ratio of homozygotes to heterozygotes (χ^2 ^= 1.02, P < 0.05). These four markers scored as dominant from ^33^P PAGE could be scored reliably for the presence of heterozygotes from the fluorescent method.

**Table 3:** Amplicon sizes used for heterozygote identification from F_2 _individuals

Primer combination	Polymorphic amplicon		Normalised to monomorphic pair
		nt	nt
^6FAM^PPT/PPT/Taq+CA	p1	261	252 and 270
^6FAM^PPT/PPT/Taq+CA	p2	274	252 and 270
^6FAM^PPT/PPT/Taq+CA	p3	307	252 and 270
^HEX^PPT/PPT/Taq+AT	p4	396	177 and 469
^HEX^PPT/PPT/Taq+AT	p5	193	177 and 469

An example of a marker that does not obviously segregate the heterozygotes from the homozygous peak present state can be seen in Figure 5B. The segregation ratio for this marker is 74:13 (present:absent) which is a small deviation from the expected 3:1 segregation ratio (χ^2 ^of 4.7, P > 0.05). The fluorescent method highlights the unreliability of this marker.

One disadvantage with this marker type is that one homozygous class will always be scored as zero. A zero result could either be a failed PCR or absence of the retrotransposon insertion, however with the multiplex amplicon pattern of SSAP a failed PCR is obvious. Monomorphic amplicons are a useful guide in the assessment of optimal PCR amplification and from this analysis are a means to determining zygosity.

The choice of monomorphic amplicons for normalisation and ratio comparisons are limited from the SSAP described here which assays the relatively low copy number, but highly polymorphic, insertion sites of the retroelement *PDR1*. In general the insertion sites of this element within the *Pisum *genus are quite diverse across the genome [1]; we have found that 2–3 amplicons per primer combination are monomorphic when comparing diverse accessions, the monomorphic number increasing as accessions become more closely related, such as between *P. sativum *cultivars.

## Conclusion

We have developed a fluorescent marker assay for SSAPs that retains the useful features of previously used ^33^P method, but has improved the accuracy of marker calling and has provided useful approximations to amplicon size. In addition it has increased the number of available markers and given the ability to recognise amplicons more sensitively and with codominant marker potential. The high-throughput method described has so far used two fluorescent tags simultaneously but there is potential for at least four, this cuts down the run cost per sample per capillary. The triplex primer method, that incorporates both labelled and unlabelled specific primers, removes the need for pre-amplifications and extensive dilution series before electrophoresis on the ABI genetic analyser. The use of software for marker peak analysis reduces the error in scoring data conferring a major benefit for genetic data analysis. Isolation of fluorescent amplicons for further characterisation and development is not immediately convenient. However here we have shown using a small scale diversity analysis that there is good transferability of the method between manual PAGE and fluorescence where band to peak patterns concur. We have also shown that using peak area values the fluorescent method can distinguish the heterozygous from homozygous classes, providing the potential for a high-throughput codominant marker system.

## Methods

### Plant material and DNA

DNA was prepared [28] from 10–15 g young leaf tissue (approx. 5–6 leaves) from the following pea accessions obtained from the JIC germplasm collection : JI15, JI73, JI281, JI399, JI813, JI1194, JI1201, JI2822, JI3253 (cv. Cameor), JI3108 (cv. Terese), JI992 (cv. Torsdag), JI2025 cv. Bohatyr. For the zygosity testing of parental accessions cv. Waverex, cv. Avola (four replicates of each), and their reciprocal F_1 _hybrids (six from each) DNA was prepared from a single leaf using an adaptation to a rapid mini-preparation method [29] modified to omit the phenol extraction as follows: a single leaf frozen with liquid N_2_, within a 1.5 ml micro-fuge tube was ground to a fine powder using a 1 ml plastic pipette tip (rounded and sealed at the tip); 400 μl of extraction buffer (500 mM NaCl, 100 mM Tris, 50 mM EDTA, pH 8.0) was added to the powder and grinding continued, followed by addition of 20 μl of 20% w/v SDS and mixed; extraction of DNA was carried out with the addition of 400 μl chloroform:isoamyl alcohol (24:1) and after thorough mixing was centrifuged for 15 min at 16,000 g; the upper aqueous phase was removed to a fresh tube and the DNA was precipitated with 800 μl of cold absolute ethanol and centrifuged again for 10 min at 16,000 g; the pelleted DNA was washed with 1 ml of 70% ethanol and then air dried, resuspended in 50 μl of TE pH 8.0 (10 mM Tris-HCl, 1 mM EDTA), stored at 4°C. The F_2 _population from the cross JI15 × JI399 has been described previously [3,26].

### Preparation of SSAP templates

A restriction digest was carried out in a 40 μl volume containing: 0.5 μg of DNA, 1 × restriction ligation (RL) reaction buffer (10 mM Tris-acetate pH 7.5, 10 mM Mg acetate, 50 mM K acetate, 5 mM DTT), 50 ng/μl BSA, 5 U *Taq*I (Invitrogen Ltd, 15218-019), incubated at 65°C for 2–3 h. Ligation of adapters was carried out as follows: to the 40 μl digest a 10 μl ligation mix containing 1 × RL buffer, 1 mM ATP, 12.5 pmol Taq adapter (Table 1), 1 U T4 DNA Ligase (Invitrogen Ltd, 15224-025) was added and the 50 μl ligation reaction was incubated at 37°C for 16 h. The restriction ligation mix was diluted to 150 μl with the addition of 100 μl of T0.1E pH 8.0 (10 mM Tris-HCl, 0.1 mM EDTA), stored at -20°C.

### Amplification of SSAP templates

The ^33^P-based SSAP PCR conditions [1], were tested by directly substituting the ^33^P labelled specific PPT primer with its fluorescently labelled counterpart as follows: 15 ng of each of the specific 6FAM or HEX labelled PPT primer (HPLC-purified) and the Taq+2 primer (Table 1) were combined in a 10 μl volume containing 1 × PCR buffer (50 mM KCl, 1.5 mM MgCl_2_, 10 mM Tris-HCl pH 8.5, 0.1 mg/ml gelatin), 200 μM each dNTP, 1 U Taq polymerase (Invitrogen Ltd, 18038-026) and 3 μl (ca. 10 ng DNA) of SSAP template (all labelled and unlabelled primers were synthesised by SIGMA-ALDRICH). A touchdown PCR cycling regime was used in all experiments: (94°C for 30 s/65°C for 30 s (reducing 0.7°C/cycle thereafter to 56°C)/72°C for 60 s) 12 cycles; (94°C for 30 s/56°C for 30 s/72°C for 60 s) 24 cycles; hold at 12°C (MJR DNA Engine). A dilution series (1/2, 1/4, 1/8) of the fluorescently labelled amplicons was made in sterile distilled water. 1 μl from the original fluorescently labelled PCR amplicons and from each of the subsequent dilutions were each added to 9 μl Hi-Di Formamide (Applied Biosystems Europe, BV 4311320) containing 0.1 μl of GENESCAN™ 1200 LIZ (Applied Biosystems Europe, AB 437525C) size standard (used in all experiments). This gave a final fluorescent amplicon dilution series of 1/10, 1/20, 1/40, 1/80 from each sample for testing on the ABI 3730 *xl *genetic analyzer, the aim being to find the optimum fluorescent conditions for marker peak analysis.

Fluorescently labelled samples were run on the Applied Biosystems 3730 *xl *as follows: POP-7™ polymer at 63°C, sample injection voltage was 1.6 kV with 15 s injection time, 8 kV run voltage for 7500 s (these conditions were used for all experiments). GeneMarker Version 1.6 software (SoftGenetics LLC^®^) was used to examine and compare peak patterns.

After the failure to match the fluorescent amplicon pattern to that of the ^33^P PAGE using the above conditions a range of primer concentrations was tested; this included the use of the specific PPT primer with and without HPLC-purification. In these experiments all of the above conditions, other than for primer content, were maintained.

### Optimisation conditions for fluorescent SSAP with the triplex PCR conditions

The conditions that were found to be optimal for the recovery of fluorescent amplicons of the expected size range and pattern as that obtained from ^33^P PAGE were as follows: 10 μl PCRs containing 0.1 μM PPT specific primer unlabelled, 0.1 μM labelled specific primer (^6FAM^PPT or ^HEX^PPT), 0.1 μM Taq + 2 primer (Table 1), all primers without HPLC purification; all other components and PCR cycling as above. The PCR samples at the 1/10 dilution of fluorescent amplicon were prepared for electrophoresis as described earlier.

### Fluorescent samples and data collection

All 16 primer combinations were tested on 12 pea lines, using the triplex reaction ^6FAM^PPT/PPT/Taq+2 and carried out in duplicate. The lines selected included, JI15, JI281, JI399, JI813, JI1194, and JI1201, the parental lines from RI (recombinant inbred) mapping populations that had been previously run with the ^33^P manual PAGE system and polymorphic markers mapped. ^HEX^PPT was used to test two primer combinations: Taq+TG, and Taq+AA for amplicon size confirmation. The F_1 _zygosity experiments with cv. Avola, cv. Waverex and their F_1 _hybrids made use of the triplex reaction with ^HEX^PPT/PPT/Taq+TG. Amplification of the 92 F_2 _individuals and parental lines from the cross JI15 × JI399 was carried out with two primer combinations: ^6FAM^PPT/PPT/Taq+CA and ^HEX^PPT/PPT/Taq+AT.

The raw data output from the ABI 3730*xl *was analysed using GeneMarker Version 1.6 software (SoftGenetics LLC^®^). For the 12 pea accessions the bin table output of peak area called by the software was transferred to an MS^® ^Excel spreadsheet. Each peak was then checked, and any missing peaks were manually called. The peak areas were converted to 1 and 0 scores as an indication of peak/marker presence and absence. Peak area tables were used directly in the F_1 _and F_2 _zygosity testing experiments, [see Additional file 1]; all calculations were carried out using MS^® ^Excel.

### Normalisation of peak areas and ratiometric analysis

In order to quantify the dosage of a *PDR1 *insertion site at a given locus in a given individual we needed to take account of the intrinsic peak area corresponding to a particular amplicon and to the amount of a sample loaded. Peak areas therefore needed to be normalised. If we consider a set of samples in which there are monomorphic bands (present in all) and polymorphic bands (either present or absent) then for all samples the ratio of the area under any pair of monomorphic bands is expected to be constant, and its variation indicates the reliability of the measure.

The ratio of the area under a polymorphic band to the area under a monomorphic band should vary according to the zygosity, and this can be used to determine allele calls. Ambiguity can be reduced by averaging the ratio with respect to several monomorphic bands.

For the F_1 _individuals ratiometric analysis was carried out using two pairs of monomorphic peaks: m1/m2 at 287/289 nt and m3/m4 at 448/470 nt [see Additional file 1]. For each replicate, four each of Avola (a) and Waverex (w), and six F_1 _hybrids from their reciprocal crosses (aw F_1_/1-F_1_/6 and wa F_1_/1-F_1_/6), monomorphic peak areas were first normalised with respect to each other, eg. m1/(m1+m2), m2/(m1+m2), m3/(m3+m4), m4/(m3+m4). Peak area ratios for each of the six F_1_s were calculated using the normalised mean of four parental (a and w) replicates, eg. aw/a, aw/w, wa/a, wa/w. Similarly each polymorphic peak area for the 12 F_1_s was first normalised with respect to either m1/m2 and m3/m4, eg, p1/(m1+m2) and p1/(m3+m4) and so forth for p2 and p3 and mean peak area ratios were calculated as for the monomorphic peaks. The means from all these calculations for the F_1 _can be seen in Table 2.

For the F_2 _individuals markers from five polymorphic fluorescent SSAP markers 261, 274, 307, 396 and 193 nt and their respective peak areas selected from two primer combinations, [see Additional file 1], were first normalised against two monomorphic peaks (Table 3) as described for the F_1_s; peak area ratios were calculated in relation to the value of the specific parental line (JI15 or JI399) that gave rise to the marker.

Plots of the peak area ratios vs raw peak area are shown in Figure 4 for the F_1 _analysis and Figures 5A and 5B for the F_2 _analysis.

## Competing interests

The authors declare that they have no competing interests.

## Authors' contributions

MK developed the manual to fluorescent method conversion conditions, cross-referenced markers from radio-labelled bands to the fluorescent peaks, collated the marker data, carried out the F_2 _data analysis, and drafted the manuscript; CM undertook the fluorescent SSAP analysis of F_1 _hybrid screen for heterozygotes, and adapted the rapid DNA leaf preparation method; JL and DB provided the expertise in the use of ABI 3730*xl *and running of fluorescent SSAP samples at JGL; NE assisted in data analysis. All authors read, edited and approved the manuscript.

## Supplementary Material

Additional file 1 **Zygosity analysis for F_1 _and F_2 _individuals**. The data provided represents raw fluorescent peak areas, the calculations for their normalisation and analysis of peak area ratios. Click here for file
